# Interim Report of the Reactogenicity and Immunogenicity of Severe Acute Respiratory Syndrome Coronavirus 2 XBB–Containing Vaccines

**DOI:** 10.1093/infdis/jiae067

**Published:** 2024-02-13

**Authors:** Spyros Chalkias, Nichole McGhee, Jordan L Whatley, Brandon Essink, Adam Brosz, Joanne E Tomassini, Bethany Girard, Darin K Edwards, Kai Wu, Arshan Nasir, Diana Lee, Laura E Avena, Jing Feng, Weiping Deng, David C Montefiori, Lindsey R Baden, Jacqueline M Miller, Rituparna Das

**Affiliations:** Infectious Disease, Research and Development, Moderna, Cambridge, Massachusetts, USA; Infectious Disease, Research and Development, Moderna, Cambridge, Massachusetts, USA; Meridian Clinical Research, Baton Rouge, Louisiana, USA; Meridian Clinical Research, Omaha, Nebraska, USA; Meridian Clinical Research, Grand Island, Nebraska, USA; Infectious Disease, Research and Development, Moderna, Cambridge, Massachusetts, USA; Infectious Disease, Research and Development, Moderna, Cambridge, Massachusetts, USA; Infectious Disease, Research and Development, Moderna, Cambridge, Massachusetts, USA; Infectious Disease, Research and Development, Moderna, Cambridge, Massachusetts, USA; Infectious Disease, Research and Development, Moderna, Cambridge, Massachusetts, USA; Infectious Disease, Research and Development, Moderna, Cambridge, Massachusetts, USA; Infectious Disease, Research and Development, Moderna, Cambridge, Massachusetts, USA; Infectious Disease, Research and Development, Moderna, Cambridge, Massachusetts, USA; Infectious Disease, Research and Development, Moderna, Cambridge, Massachusetts, USA; Department of Surgery, Duke University Medical Center, Durham, North Carolina, USA; Division of Infectious Diseases, Brigham & Women's Hospital, Boston, Massachusetts, USA; Infectious Disease, Research and Development, Moderna, Cambridge, Massachusetts, USA; Infectious Disease, Research and Development, Moderna, Cambridge, Massachusetts, USA

**Keywords:** mRNA vaccine, SARS-CoV-2 variants, COVID-19, Omicron XBB.1.5, JN.1

## Abstract

**Background:**

Monovalent Omicron XBB.1.5–containing vaccines were approved for coronavirus disease 2019 (COVID-19) 2023–2024 immunizations.

**Methods:**

This ongoing, open-label, phase 2/3 study evaluated messenger RNA (mRNA)-1273.815 monovalent (50-µg Omicron XBB.1.5 spike mRNA) and mRNA-1273.231 bivalent (25-µg each Omicron XBB.1.5 and BA.4/BA.5 spike mRNAs) vaccines, administered as fifth doses to adults who previously received primary series, third doses of an original mRNA COVID-19 vaccine, and fourth doses of an Omicron BA.4/BA.5 bivalent vaccine. Interim safety and immunogenicity 29 days after vaccination are reported.

**Results:**

Participants (randomized 1:1) received 50-µg of mRNA-1273.815 (n = 50) or mRNA-1273.231 (n = 51); median intervals (interquartile range) from prior BA.4/BA.5 bivalent doses were 8.2 (8.1–8.3) and 8.3 (8.1–8.4) months, respectively. Fold increases in neutralizing antibody (nAb) against the severe acute respiratory syndrome coronavirus 2 (SARS-CoV-2) variants from prebooster nAb levels were numerically higher against XBB.1.5, XBB.1.16, EG.5.1, BA.2.86, and JN.1 than BA.4/BA.5, BQ.1.1, or D614G on day 29. Monovalent vaccine also cross-neutralized FL.1.5.1, EG.5.1, BA.2.86, HK.3.1, HV.1, and JN.1 variants in a participant subset (n = 20) 15 days after vaccination. Reactogenicity was similar to that of mRNA-1273 vaccines.

**Conclusions:**

XBB.1.5-containing mRNA-1273 vaccines elicit robust, diverse nAb responses against more recent SARS-CoV-2 variants, including JN.1, supporting the XBB.1.5-spike update for COVID-19 vaccines.

## Teaser

XBB.1.5-containing messenger RNA-1273 vaccines administered as fifth doses to adults elicited potent and diverse neutralizing responses against Omicron XBB and other recent severe acute respiratory syndrome coronavirus 2 variants, supporting authorization and approval of monovalent XBB.1.5-containing vaccines for 2023-2024 immunizations.

Given the continuous viral evolution and emergence of severe acute respiratory syndrome coronavirus 2 (SARS-CoV-2) variants with the potential to evade immunity provided by prior vaccination and/or infection, health agencies have recommended updates of coronavirus disease 2019 (COVID-19) vaccines to match circulating variants as closely as possible [1–4]. Bivalent booster vaccines comprising messenger RNAs (mRNAs) encoding the original SARS-CoV-2 and Omicron BA.1 (mRNA-1273.214) or BA.4/BA.5 (mRNA-1273.222) variants were deployed globally during 2022–2023 to prevent COVID-19 caused by SARS-CoV-2 Omicron variants [1, 3]. In clinical studies, these bivalent vaccines demonstrated increased neutralizing antibody (nAb) responses against the matched BA.1 and BA.4/BA.5 variants [5–8], with relatively lower titers against divergent variants not contained in the vaccines (eg, BQ.1.1, XBB.1.5, and XBB.1.16) [5–7]. In addition, real-world data in the United States has shown that the BA.4/BA.5-containing bivalent mRNA-boosters provided enhanced protection against COVID-19 compared with individuals who did not receive the BA.4/BA.5 bivalent vaccine [1, 3, 9–13].

With the emergence of Omicron XBB variants and their ability to escape preexisting immunity, XBB variant–containing boosters were evaluated for use in 2023–2024 COVID-19 immunizations [14, 15]. This phase 2/3, open-label study assessed the safety and immunogenicity of mRNA-1273 XBB-1.5–containing booster vaccines administered as fifth doses to adults who previously received a 2-dose primary series, a third dose of an original mRNA COVID-19 vaccine, and a fourth dose of a bivalent vaccine (Omicron BA.4/BA.5 plus original strain). Participants were randomized 1:1 to receive 50-µg doses of mRNA-1273.815 monovalent (50-µg Omicron XBB.1.5 spike mRNA) or mRNA-1273.231 bivalent (25-µg Omicron XBB.1.5 and 25-µg Omicron BA.4/BA.5 spike mRNAs) vaccines. The results of an interim safety and immunogenicity analysis 1 month after vaccination are reported herein.

## METHODS

### Study Design and Participants

This open-label, ongoing phase 2/3 study (clinicaltrials.gov NCT04927065) evaluates the immunogenicity of SARS-CoV-2 variant–containing mRNA-1273 vaccine candidates against COVID-19. Part J of the study evaluates the reactogenicity, adverse events (AEs) and immunogenicity of 50-µg doses of the monovalent mRNA-1273.815 vaccine (50-µg Omicron XBB.1.5 spike protein mRNA) and bivalent mRNA-1273.231 vaccine (25-µg Omicron XBB.1.5 and 25-µg Omicron BA.4/BA.5 spike protein mRNAs). The vaccines were administered as fifth doses to adults who previously received a 2-dose primary series, an immunogenicity booster dose of an original COVID-19 vaccine, and a booster dose of a bivalent (original plus Omicron BA.4/BA.5) vaccine. Interim 15- and 29-day analysis results are reported.

Adults with a known history of SARS-CoV-2 infection ≤3 months from screening were excluded from the study. Additional details of inclusion/exclusion criteria are provided in the Supplementary Materials.

### Trial Oversight

The trial is being conducted across 9 US sites, in accordance with the International Council for Harmonisation of Technical Requirements for Registration of Pharmaceuticals for Human Use, Good Clinical Practice guidelines, and human experimentation guidelines of the US Department of Health and Human Services and/or those of the authors’ institutions and in accordance with the ethical standards of the Helsinki Declaration (1964; amended most recently in 2013). The Central Institutional Review Board (Columbia, Maryland) approved the protocol and consent forms. All participants provided written informed consent.

### Trial Vaccine

The monovalent mRNA-1273.815 50-µg vaccine (Spikevax; Moderna) contains 50 μg of mRNA encoding the prefusion-stabilized spike glycoprotein of SARS-CoV-2 variants XBB.1.5/XBB.1.9.1. The bivalent mRNA-1273.231 50-µg vaccine contains 25 µg each of mRNAs encoding the prefusion-stabilized spike glycoproteins of the SARS-CoV-2 Omicron variant (BA.4/BA.5) and the SARS-CoV-2 variants XBB.1.5/XBB.1.9.1, mixed in a 1:1 ratio. In both vaccines, mRNAs were encapsulated in lipid nanoparticles, as described elsewhere [16]. The booster doses of mRNA-1273.815 and mRNA-1273.231 were each administered by intramuscular injection at 50 µg of mRNA.

### Objectives

The primary objectives of the study are evaluation of safety including reactogenicity and unsolicited AEs of mRNA-1273.815 and mRNA-1273.231 when administered as a fifth (third booster) dose. The evaluation of immunogenicity based on nAb responses against Omicron BA.4/BA.5 and XBB.1.5 variants contained in the vaccines and the predominant BQ.1.1 variant circulating at the time were also primary objectives at days 15 and 29. The incidence of symptomatic and asymptomatic SARS-CoV-2 infections was a prespecified, exploratory end point. SARS-CoV-2 infections and COVID-19 events were actively surveilled through weekly contact and blood sampling throughout the study (Supplementary Methods).

### Randomization

Approximately 100 participants were planned to be randomized in a 1:1 ratio to receive a single booster dose of mRNA-1273.815 (50 µg) or mRNA-1273.231 (50 µg). The 2 groups were randomized 1:1 in an open-label manner.

### Immunogenicity Assessment

The nAb titers for the BA.4/BA.5 and ancestral SARS-CoV-2 (D614G) variants were evaluated using validated lentivirus-based pseudovirus neutralizing assays, and those for XBB.1.5, XBB.1.16, BQ.1.1, EG.5.1, BA.2.86, and JN.1 using qualified lentivirus-based pseudovirus neutralizing assays (Duke University) [17]. In a separate analysis to enable rapid assessment of more recently circulating variants, ancestral SARS-CoV-2 (Wuhan-Hu-1 [D614G]) and BA.4/BA.5, XBB.1.5, XBB.1.16, XBB.2.3.2, EG.5.1, FL.1.5.1, BA.2.86, HK.3.1, HV.1, and JN.1 variants were also assessed using a research grade vesicular stomatitis virus (VSV)–based SARS-CoV-2 pseudovirus neutralization assay (Supplementary Methods).

### Statistical Analysis

Safety was assessed in the safety set and solicited local and systemic adverse reactions (ARs) in the solicited safety set; assessments included solicited ARs ≤7 days and unsolicited AEs ≤28 days after booster administration and serious AEs, AEs leading to discontinuation from study vaccine and/or participation, medically attended AEs, and AEs of special interest from day 1 through the interim study period.

Immunogenicity against prespecified variants (BA.4/BA.5, XBB.1.5, and BQ.1.1) and XBB.1.16 (an emerging variant) as well as ancestral SARS-CoV-2 (D614G) were assessed prebooster and at days 15 and 29 after the booster dose, and against EG.5.1, BA.2.86, and JN.1 variants prebooster and at day 29 after the booster dose. Immunogenicity was assessed in all participants in the per-protocol immunogenicity set (those with and without prior SARS-CoV-2 infection) and in those with or without SARS-CoV-2 infection before the booster (absence of infection demonstrated by negative binding antibody against SARS-CoV-2 nucleocapsid and negative reverse-transcription polymerase chain reaction results on day 1). The study was designed with approximately 100 evaluable participants to rapidly obtain clinical data for the fall 2023 vaccines. The study size was not powered to compare the immune responses of the 2 XBB.1.5-containing vaccines, as it was anticipated that the responses would be similar. No statistical hypothesis testing with respect to the immune responses was performed, and results are descriptive, as a larger randomized trial would be needed to adequately power the study to compare the noninferiority of the vaccines (mRNA-1273.815 vs mRNA-1273.231). The nAb geometric mean (GM) levels and the GM fold rises (GMFRs) from prebooster baseline levels after the mRNA-1273.815 and mRNA-1273.231 doses are provided with the corresponding 95% confidence intervals.

To assess emerging variants more rapidly, a separate analysis of variants that emerged after study initiation and were not prespecified in the protocol was performed in a randomly selected subgroup of participants (n = 20) who received mRNA-1273.815, including ancestral SARS-CoV-2 (Wuhan-Hu-1 [D614G]) and BA.4/BA.5, XBB.1.5, XBB.1.16, XBB.2.3.2, EG.5.1, FL.1.5.1, BA.2.86, HK.3.1, HV.1, and JN.1 variants.

Symptomatic and asymptomatic SARS-CoV-2 infections were assessed as an exploratory end point, starting 14 days after the booster dose. Descriptive summaries of any occurrences were to be provided for each study arm. The data cutoff date for day 29 interim analysis was 25 May 2023. All analyses were conducted using SAS software, version 9.4 or higher.

## RESULTS

In April 2023, 101 participants received the monovalent mRNA-1273.815 (n = 50) and bivalent mRNA-1273.231 (n = 51) vaccines. Baseline characteristics were generally balanced for the groups (Table 1). Mean ages were 51.6 years in the mRNA-1273.815 and 48.4 years in the mRNA-1273.231 groups, and 60% and 61% of participants, respectively, were female. The majority of participants were white (90.0% and 80.4%, respectively), 8.0% and 7.8% were black or African American, and most were of non-Hispanic or non-Latino ethnicity (80.0% and 86.3%, respectively). The percentages of participants with evidence of SARS-CoV-2 infection before the booster were 68.0% and 78.4%, respectively, in the mRNA-1273.815 and mRNA-1273.231 groups.

**Table 1.** Demographics and Characteristics of Study Participants (Safety Set)

​ Characteristic	Participants , No. (%)^a^	
	Monovalent mRNA-1273.815 XBB.1.5 (n = 50​)	Bivalent mRNA-1273.231 XBB.1.5 + BA.4/BA.5 ​(n = 51​)
Age, y		
Mean (SD)	51.6​ (15.2)	48.4​ (15.2)
Median (range)	54.5 (21–84)​	48.0 (24–82)​
Age ≥65 y	11 (22.0)​	7 (13.7)​
Sex		
Male	20 (40.0)	20 (39.2)
Female​	30 (60.0)​	31 (60.8)​
Race		
White	45 (90.0)	41 (80.4)
Black/African American​	4 (8.0)​	4 (7.8)​
Asian	1 (2.0)	3 (5.9)
Multiracial or other	0	3 (5.9)
Ethnicity		
Hispanic or Latino	9 (18.0)	6 (11.8)
Not Hispanic or Latino	40 (80.0)	44 (86.3)
Not reported	1 (2.0)	1 (2.0)
BMI, mean (SD)^b^	30.57 (8.20)	31.42 (8.02)
Interval between doses, median (IQR), mo		
Between 2nd and 3rd doses	8.2 (7.8–9.8)​	9.0 (7.7–11.5)​
Between 3rd and 4th doses	9.8 (8.3–10.3)​	9.2 (8.2–10.3)​
Between 4th and 5th doses	8.2 (8.1–8.3)​	8.3 (8.1–8.4)​
Prior SARS-CoV-2 infection​^c^	34 (68.0)​	40 (78.4)​

Abbreviations: BMI, body mass index; IQR, interquartile range; mRNA, messenger RNA; SARS-CoV-2, severe acute respiratory syndrome coronavirus 2; SD, standard deviation.

^a^Data represent no. (%) of participants unless otherwise specified. Percentages are based on the number of participants in the safety set, and they may not total 100% owing to rounding.

^b^BMI calculated as weight in kilograms divided by height in meters squared.

^c^The prior SARS-CoV-2 status was positive if there was evidence of previous SARS-CoV-2 infection, defined as positive binding antibody against the SARS-CoV-2 nucleocapsid or positive reverse-transcription polymerase chain reaction result.

The median (interquartile range [IQR]) intervals were 8.2 (7.8–9.8) and 9.0 (7.7–11.5) months between the second doses of the primary series and third doses of an original COVID-19 booster vaccine, and 9.8 (8.3–10.3) and 9.2 (8.2–10.3) months between the booster (third) doses of an original vaccine and booster (fourth) dose of a BA.4/BA.5-containing bivalent vaccine in the mRNA-1273.815 and mRNA-1273.231 groups, respectively. The median (IQR) intervals between the prior (fourth) and the fifth doses of the BA.4/BA.5-containing bivalent vaccine were 8.2 (8.1–8.3) months for the mRNA-1273.815 and 8.3 (8.1–8.4) months for the mRNA-1273.231 group [7].

**Figure 1.**
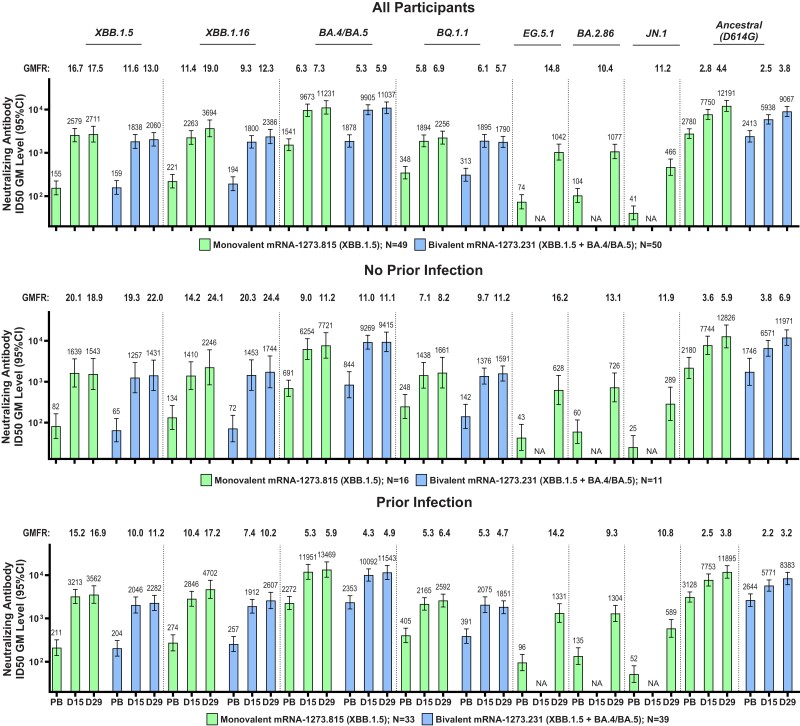
Neutralizing antibodies (nAbs) after a booster dose of XBB.1.5-containing monovalent and bivalent vaccines against XBB.1.5, XBB.1.16, BA.4/BA.5, BQ.1.1, EG.5.1, BA.2.86, and JN.1 variants and ancestral severe acute respiratory syndrome coronavirus 2 (SARS-CoV-2) (D614G). Geometric mean (GM) levels for nAb titers were assessed before the booster (prebooster [PB]) and at days 15 (D15) and 29 (D29) after vaccination for the monovalent (messenger RNA [mRNA]-1273.815) and bivalent (mRNA-1273.231) vaccines using a pseudovirus assay, as described elsewhere [5, 6] and in the Supplementary Methods. Analyses were performed in all participants in the per-protocol set for immunogenicity, regardless of prior SARS-CoV-2 infection, and in those without or with prior infection. Antibody values reported as below the lower limit of quantification (LLOQ; 18.5 [1.3 log_10_] for ancestral SARS-CoV-2 [D614G] and 36.7 [1.6 log_10_] for Omicron BA.4/BA.5) are replaced by 0.5 × LLOQ. Values above the upper limit of quantification (ULOQ; 45 118 [4.7 log_10_] for ancestral SARS-CoV-2 [D614G] and 13 705 [4.1 log_10_] for Omicron BA.4/BA.5) are replaced by the ULOQ if actual values are not available. Antibody values reported as below the lower limit of detection (LOD; 10 for XBB.1.5, XBB.1.16, BQ.1.1, EG.5.1, BA.2.86, and JN.1) are replaced by 0.5 × LOD. Abbreviations: GMFR, GM fold rise at D15 and D29 relative to PB levels; ID_50_, median infective dose; NA, not available (D15 data for EG.5, BA.2.86, and JN.1).

Immune responses were assessed against XBB.1.5, XBB.1.16, BQ.1.1, and BA.4/BA.5 variants and ancestral SARS-CoV-2 (D614G), at days 15 and 29 with both vaccines, and at day 29 for EG.5.1, BA.2.86, and JN.1 variants with the monovalent vaccine. The prebooster nAb GM levels were lower against the XBB.1.5, XBB.1.16, and BQ.1.1 variants than against the BA.4/BA.5 variant and ancestral SARS-CoV-2 (D614G) for both mRNA-1273.815 and mRNA-1273.231 vaccines, and were lowest for EG.5.1, BA.2.86, and JN.1 after the monovalent vaccine (Figures 1, and Supplementary Figures 1 and 2, and Supplementary Table 2).

Both vaccines increased nAb GM levels from prebooster levels against all variants. The nAb GM levels as well as GMFRs from prebooster baseline levels were generally higher at day 29 than at day 15 and were numerically higher after the monovalent booster than after the bivalent booster against XBB.1.5, XBB.1.16, BQ.1.1, and ancestral SARS-CoV-2 (D614G) and comparable against BA.4/BA.5 variants for both vaccines. The GMFRs from baseline were higher against the XBB.1.5, XBB.1.16, EG.5.1, BA.2.86, and JN.1 variants than against the BA.4/BA.5 and BQ.1.1 variants and ancestral SARS-CoV-2 (D614G). Findings were similar regardless of prebooster infection status for both vaccines across variants (Figure 1 and Supplementary Table 3). Among younger participants, nAb levels were comparable for the 2 vaccines, and in older participants, the monovalent vaccine elicited higher titers versus the bivalent vaccine; however, the group sizes limited overall interpretation (Supplementary Table 4).

To rapidly assess neutralization induced by the monovalent vaccine against emerging variants, the day 15 responses of a randomly selected subset of participants (n = 20) who received the monovalent vaccine against XBB.2.3.2, EG.5.1, FL.1.5.1, BA.2.86-v2, HK.3.1, HV.1, and JN.1 were assessed using a VSV-based SARS-CoV-2 pseudovirus neutralization assay (Supplementary Figure 3) [15]. The results of this assay were similar to those of the lentivirus assay, including the baseline prebooster levels against the variants and ancestral SARS-CoV-2 (D614G), with the lowest baselines seen against the JN.1, HK.3.1 and HV.1 variants. The GMFRs from baseline against the XBB.1.5, XBB.1.16, EG.5.1, and BA.2.86 variants were also higher than those of the BA.4/BA.5 variants and ancestral SARS-CoV-2 (D614G). In addition, the FL.1.5.1, BA.2.86-v2, HK.3.1, HV.1, and JN.1 variants tested in this assay also exhibited GMFRs higher than those of the BA.4/BA.5 variants and ancestral SARS-CoV-2 (D614G).

The incidences of symptomatic SARS-CoV-2 infection and asymptomatic SARS-CoV-2 infection 14 days after the booster were evaluated as an exploratory end point. Up to the study cutoff date, no COVID-19 events were detected in either vaccine group.

**Figure 2.**
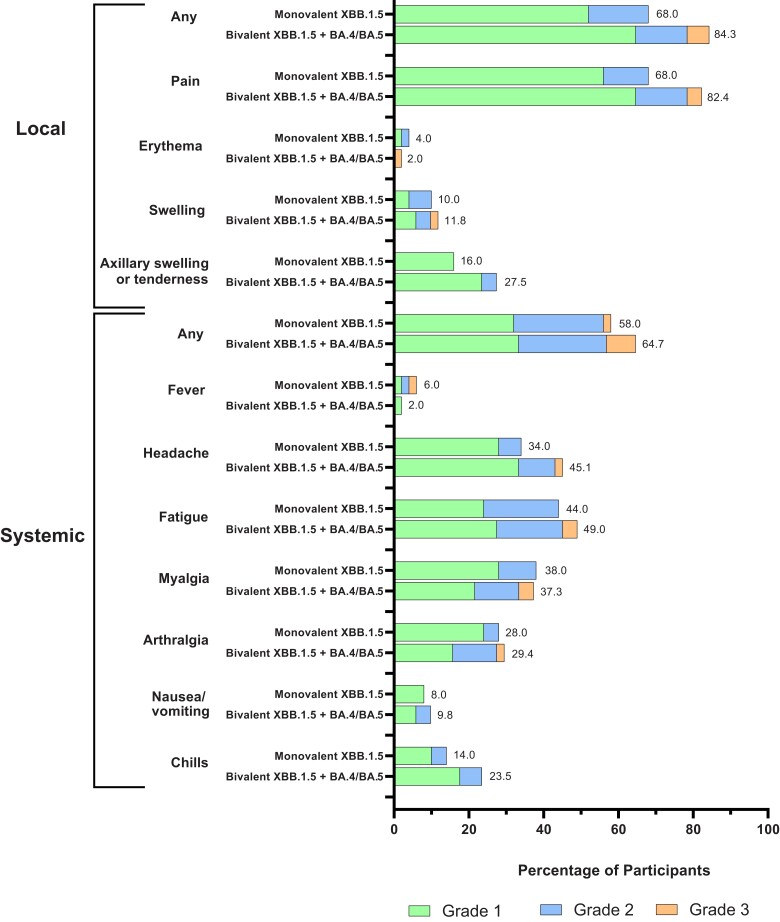
Solicited local and systemic adverse reactions. Shown are the percentages of participants in whom solicited local or systemic adverse reactions occurred within 7 days after the messenger RNA (mRNA) booster dose in the solicited safety set (n = 50 in the monovalent XBB.1.5 vaccine group and n = 51 in the bivalent XBB.1.5 vaccine group).

During a median follow-up (IQR) of 29 (29–30) days in this interim analysis of the planned 181 day follow-up period, the occurrence of solicited local and systemic ARs and unsolicited AEs in both vaccine groups were overall similar to those reported elsewhere for the original mRNA-1273 50-µg and Omicron BA.4/BA.5–containing bivalent mRNA-1273 vaccines [5, 6]. The frequencies of solicited ARs were comparable in the bivalent-mRNA-1273.231 (45 [88.2%]) and monovalent-mRNA-1273.815 (38 [76.0%]) groups, and the majority of ARs were grade 1–2 events, with no grade 4 events reported to date in this interim analysis (Figure 2 and Supplementary Table 5). The occurrences of unsolicited AEs were also similar in the bivalent (9 [17.6%]) and monovalent (6 [12.0%]) group within 28 days after injection (Supplementary Table 6). There were no serious or fatal AEs or AEs leading to study discontinuation. No additional AEs were reported through the data cutoff date in this interim analysis.

## DISCUSSION

SARS-CoV-2 Omicron XBB.1.5–containing monovalent and bivalent vaccines elicited potent and diverse neutralizing responses against Omicron XBB-lineage variants, including XBB.1.5 and XBB.1.16, as well as the divergent variants EG.5.1, FL.1.5.1, BA.2.86, HK.3.1, HV.1, and JN.1 [4, 18–22] in an interim analysis of the present study [5–7]. The nAb responses overall were numerically higher for the monovalent than for the bivalent vaccine against the XBB variants, and although the prevaccination titers for BA.4/BA.5 and D614G were higher than for other variants, the postvaccination responses were increased against all variants tested, indicating an ability to neutralize variants despite antigenic divergences from XBB-1.5. The diversity and magnitude of the antibody response suggests an ability to broaden the antibody repertoire, despite potential antigenic imprinting from prior infection or vaccination [23].

The immune responses following both vaccines were comparable against the XBB.1.5, XBB.1.16, and XBB.2.3.2 variants, consistent with the limited antigenic differences among these 3 variants [18, 19]. Notably the XBB-containing vaccines cross-neutralized the EG.5.1, FL.1.5.1, BA.2.86, HK.3.1, HV.1, and JN.1 variants [4, 18–20], suggesting that the monovalent mRNA-1273.815 vaccine has the potential to provide protection against these emerging variants. The EG.5.1, FL.1.5.1, HK.3.1, and HV.1 variants are descendants of the Omicron XBB-lineage, with a similar genetic makeup to that of XBB.1.5, and contain an additional spike mutation (F456L) [4]. EG.5.1, HV.1, HK.3.1, and FL1.5.1 surged in the United States in 2023 [19, 21]. The BA.2.86 variant, detected globally, carries multiple additional mutations beyond those seen in the spike Omicron spike protein [4, 20, 22], and the JN.1 (BA.2.86.1.1) variant possesses an additional spike mutation, L455S, compared with BA.2.86, with a markedly increased prevalence [22, 24]. Despite these antigenic differences, the data herein indicate that the XBB.1.5 monovalent vaccine can elicit a potent response against multiple variants that circulated in 2023, including the more recent JN.1 variant. Based on these results, the monovalent XBB-1.5-containing vaccine is expected to provide protection against COVID-19 due to recent circulating variants, including JN.1.

The present study has several limitations, as it was not designed to evaluate vaccine efficacy and was not powered for the immunogenicity objective owning to the need to rapidly evaluate the vaccine candidates in the clinic per regulatory guidance [25]. Although nAb levels are correlative with vaccine efficacy, a correlate of protection has not been formally established, and the end points in this study have not been validated to predict protection against COVID-19 [17, 26]. Nonetheless, the magnitude of the antibody responses to the XBB.1.5-containing boosters observed in the study might be correlate with reduced COVID-19 risk [17, 26]. Evaluation of antibody persistence is ongoing in the present study, and although the percentage of participants with prior SARS-CoV-2 infection was higher in the bivalent vaccine group, there was no observed bias toward increased neutralization capacity in the bivalent vaccine group. The rapid evaluation of neutralization in a subset of participants for specific variants was performed using a different assay than that used for the entire study population and time points; the 2 assays overall generate similar results [15].

The reactogenicity of the XBB.1.5-containing vaccines appears to be similar to that of the original and other variant-containing vaccines, and longer-term follow-up of the study participants is ongoing. The frequencies of the prespecified primary end points for serious AEs, medically attended AEs, AEs leading to withdrawal, and AEs of special interest through the study end (day 181) are reported 29 days after vaccination in this interim analysis of the planned 181-day follow-up period; longer-term evaluation of these end points through study completion continues in the ongoing study. The study participants, volunteers who were selected on the basis of prespecified criteria for prior vaccine and booster doses, and who self-reported AEs, may differ from the general population. Thus, the frequency of AEs observed in this study may not entirely coincide with real-world use of the vaccine; however, the results herein are consistent with those previously reported for monovalent and bivalent mRNA-1273 vaccines, including real-world evidence [5, 6, 9, 11, 12, 27, 28].

In summary, these interim results indicate that the monovalent XBB.1.5–containing vaccine can elicit potent and broad neutralizing responses against circulating SARS-CoV-2 variants, including JN.1. The interim results of this study support the strategy of updating COVID-19 vaccines to match antigenically divergent variants and support the selection of the XBB.1.5 spike sequence for the 2023–2024 vaccine update [2].

## Supplementary Data

Supplementary materials are available at *The Journal of Infectious Diseases* online (http://jid.oxfordjournals.org/). Supplementary materials consist of data provided by the author that are published to benefit the reader. The posted materials are not copyedited. The contents of all supplementary data are the sole responsibility of the authors. Questions or messages regarding errors should be addressed to the author.

## Supplementary Material

jiae067_Supplementary_Data

## Notes

***Acknowledgments.*** We thank the participants in the trial and the members of the mRNA-1273 trial team (listed in the Supplementary Materials) for their dedication and contributions to the trial; the Immune Assay Team at Duke University Medical Center, Durham, North Carolina, for pseudovirus neutralization antibody assays; Daniela Montes Berrueta and Matthew Koch from Moderna for the VSV SARS-CoV-2 pseudovirus analyses and data; Michael Whitt and Rita Kansal from the University of Tennessee Health Science Center, Memphis, for generating the VSV-based SARS-CoV-2 pseudoviruses; and Frank J. Dutko (Moderna consultant) for figure development and editorial support.

***Data sharing.*** As the trial is ongoing, access to patient-level data and supporting clinical documents with qualified external researchers will be made available on request and subject to review after trial completion (November 2024). A materials transfer and/or data access agreement with the sponsor will be required for accessing shared data. Such requests can be made to the correspondence author.

***Financial support***. This work was supported by Moderna, Inc. Funding to pay the Open Access publication charges for this article was provided by Moderna, Inc.
