# Correction to: Interim Report of the Reactogenicity and Immunogenicity of Severe Acute Respiratory Syndrome Coronavirus 2 XBB–Containing Vaccines

**DOI:** 10.1093/infdis/jiae186

**Published:** 2024-04-18

After the original publication of this article [Chalkias S, McGhee N, Whatley JL et al. Interim Report of the Reactogenicity and Immunogenicity of Severe Acute Respiratory Syndrome Coronavirus 2 XBB–Containing Vaccines. *The Journal of Infectious Diseases,* jiae067, https://doi.org/10.1093/infdis/jiae067] it was discovered that Figure 1 was missing key data. Figure 1 has been updated in the version currently available online.

