# Supplementary material for: Interim Report of the Reactogenicity and Immunogenicity of Severe Acute Respiratory Syndrome Coronavirus 2 XBB–Containing Vaccines
Source: J Infect Dis. 2024 Feb 13;230(2):e279–86. doi: 10.1093/infdis/jiae067 (PMC11326827; doi:10.1093/infdis/jiae067)
Supplement: jiae067_Supplementary_Data [file jiae067_supplementary_data.zip › Chalkias_JID78788R1_Supplement_Revised_Jan_26_Clean.docx]

# **Supplement**

## **List of Investigators**

| **Investigator** | **Institution** | **Location** |
| --- | --- | --- |
| Adam Brosz | Meridian Clinical Research | Grand Island, Nebraska |
| Laurence Chu | Benchmark Research | Austin, Texas |
| Michael Cotugno | Benchmark Research | Metairie, Louisiana |
| Brandon Essink | Meridian Clinical Research | Omaha, Nebraska |
| Carlos Fierro | Johnson County Clin-Trials | Lenexa, Kansas |
| Carl Griffin | Lynn Health Science Institute | Oklahoma City, Oklahoma |
| Greg Hachigian | Benchmark Research | Sacramento, California |
| Charles Harper. Keith Vrbicky, | Meridian Clinical Research | Norfolk, Nebraska |
| Jordan Whatley | Meridian Clinical Research | Baton Rouge, Louisiana |

## **Supplementary methods**

***Part J Study Eligibility Criteria***

*Inclusion Criteria*

Each participant must meet all of the following criteria to be enrolled in this study:

1. Male or female, at least 18 years of age at the time of consent (Screening Visit).
2. Investigator’s assessment that participant understands and is willing and physically able to comply with protocol-mandated follow-up, including all procedures.
3. Participant has provided written informed consent for participation in this study, including all evaluations and procedures as specified in this protocol.
4. Female participants of nonchildbearing potential may be enrolled in the study. Nonchildbearing potential is defined as surgically sterile (history of bilateral tubal ligation, bilateral oophorectomy, hysterectomy) or postmenopausal (defined as amenorrhea for ≥12 consecutive months prior to screening [Day 0] without an alternative medical cause). A follicle-stimulating hormone (FSH) level may be measured at the discretion of the investigator to confirm postmenopausal status.
5. Female participants of childbearing potential may be enrolled in the study if the participant fulfills all of the following criteria:
   - Has a negative pregnancy test on the day of vaccination (Day 1).
   - Has practiced adequate contraception (Section [10.3](#_bookmark158)) or has abstained from all activities that could result in pregnancy for at least 28 days prior to Day 1.
   - Has agreed to continue adequate contraception through 3 months following vaccination.
   - Is not currently breastfeeding.

Adequate female contraception is defined as consistent and correct use of a United States FDA-approved contraceptive method in accordance with the product label (Section [10.3](#_bookmark158)).

1. Participant must have been either previously enrolled in the mRNA-1273-P301 (COVE) study, must have received 2 doses of mRNA-1273 in that study, with his/her second dose at least 6 months prior to enrollment in mRNA-1273-P205, and must be currently enrolled and compliant in that study (ie, has not withdrawn or discontinued early); or participant must have received 2 doses of mRNA-1273 under the EUA with their second dose at least 6 months prior to enrollment in mRNA-1273-P205; or have received a 2 dose primary series of mRNA-1273 followed by a 50 µg booster dose of mRNA-1273 in the mRNA-1273-P301 (COVE) study or under EUA at least 3 months prior to enrollment in mRNA-1273-P205; and able to provide proof of vaccination status at the time of

screening (Day 1); or for enrollment in Part A.2, participant must be currently enrolled and compliant in Part A.1 of the mRNA-1273-P205 study and must have received their first booster dose of mRNA-1273.211 50 µg; or for enrollment in Part J, participant must meet at least one of the following criteria:

- - Completed enrollment in Part H of the mRNA-1273-P205 study; or
  - Received a 2-dose primary series of mRNA-1273 (100 µg) followed by a 50 µg booster dose of mRNA-1273 in the mRNA-1273-P301 (COVE) study or under EUA, followed by a 50 µg booster dose of mRNA-1273.222 under EUA at least 3 months prior to enrollment in Part J of mRNA-1273-P205; or
  - Previously received a 2-dose primary series of mRNA vaccine against SARS-CoV-2 followed by a booster dose of a monovalent mRNA vaccine, followed by a second booster dose of a bivalent mRNA vaccine

Participants in Part J must also provide proof of vaccination status at the time of screening (Day 0 or Day 1).

*Exclusion Criteria*

Participants meeting any of the following criteria at the Screening Visit, unless noted otherwise, were excluded from the study:

1. Had significant exposure to someone with SARS-CoV-2 infection or coronavirus disease 2019 (COVID-19) in the past 14 days, as defined by the CDC as a close contact of someone who has had COVID-19.
2. Has known history of SARS-CoV-2 infection within 3 months prior to enrollment.
3. Is acutely ill or febrile (temperature ≥ 38.0°C/[100.4°F]) less than 72 hours prior to or at the Screening Visit or Day 1. Participants meeting this criterion may be rescheduled and will retain their initially assigned participant number.
4. Currently has symptomatic acute or unstable chronic disease requiring medical or surgical care, to include significant change in therapy or hospitalization for worsening disease, at the discretion of the investigator.
5. Has a medical, psychiatric, or occupational condition that may pose additional risk as a result of participation, or that could interfere with safety assessments or interpretation of results according to the investigator’s judgment.
6. Has a current or previous diagnosis of immunocompromising condition to include human immunodeficiency virus, immune-mediated disease requiring immunosuppressive treatment, or other immunosuppressive condition.
7. Has received systemic immunosuppressants or immune-modifying drugs for > 14 days in total within 6 months prior to screening (for corticosteroids ≥ 10 mg/day of prednisone equivalent) or is anticipating the need for immunosuppressive treatment at any time during participation in the study.
8. Has known or suspected allergy or history of anaphylaxis, urticaria, or other significant AR to the vaccine or its excipients.
9. Has a documented history of myocarditis or pericarditis within 2 months prior to Screening Visit (Day 0).
10. Coagulopathy or bleeding disorder considered a contraindication to intramuscular (IM) injection or phlebotomy.
11. Has received or plans to receive any licensed vaccine ≤ 28 days prior to the injection (Day 1) or a licensed vaccine within 28 days before or after the study injection, with the exception of influenza vaccines, which may be given 14 days before or after receipt of a study vaccine.
12. Has received systemic immunoglobulins or blood products within 3 months prior to the Screening Visit (Day 0) or plans for receipt during the study.
13. Has donated ≥ 450 mL of blood products within 28 days prior to the Screening Visit or plans to donate blood products during the study.
14. Plans to participate in an interventional clinical trial of an investigational vaccine or drug while participating in this study.
15. Is an immediate family member or household member of study personnel, study site staff, or Sponsor personnel.
16. Is currently experiencing an SAE in Study mRNA-1273-P301 (COVE) at the time of screening for this study.

**Sample Size Determination**

As there was no formal prespecified hypothesis testing in part J, sample size was not driven by statistical assumptions. The number of proposed participants was considered sufficient to provide a descriptive summary of the safety and immunogenicity of each treatment arm. The study was designed with a need to rapidly obtain clinical data for the fall vaccines in support of regulatory requests. The target enrollment planned was approximately 100 participants to be randomized in a 1:1 ratio to receive a single booster dose of either mRNA-1273.815 (50 µg) or mRNA-1273.231 (50 µg).

### **Assessment of SARS-CoV-2 Infection**

SARS-CoV-2 infection was actively surveilled through weekly contact and blood draws. SARS-CoV-2 infection was a combination of symptomatic infection (COVID-19) and asymptomatic SARS-CoV-2 infection for participants with negative SARS-CoV-2 status pre-booster. Symptomatic infection was evaluated using the primary case definition in the COVE study^25,37^ and a secondary case definition based on the Centers for Disease Control and Prevention (CDC) criteria.^38^ Asymptomatic SARS-CoV-2 infection was defined as a positive reverse-transcriptase polymerase chain reaction (RT-PCR) test or a positive serologic test for anti-nucleocapsid antibody after a negative test at the time of enrollment, in the absence of symptoms. Descriptive summaries of symptomatic COVID-19 disease, asymptomatic SARS-CoV-2 infection, as well as COVID-19 regardless of symptoms were provided for each study arm.

### **Immunogenicity Assays**

***Lentivirus-Based SARS-CoV-2 Spike-Pseudotyped Virus Neutralization Assay***

SARS-CoV-2 neutralizing antibodies (nAb) in samples were assessed using a SARS-CoV-2 Spike (S)-Pseudotyped Virus Neutralization Assay (PsVNA) in 293/ACE2 cells.^1^ The PsVNA quantifies nAb using lentivirus particles that express full-length spike proteins on their surface, and contain a firefly luciferase reporter gene for quantitative measurements of infection in transduced 293T cells expressing high levels of ACE2 (293T/ACE2 cells) by relative luminescence units (RLU). Serial dilution of antibodies was used to produce a dose-response curve. Neutralization was measured as the serum dilution at which RLU was reduced by 50% (ID50) relative to mean RLU in virus control wells (cells + virus but no sample) after subtraction of mean RLU in cell control wells (cells only). Positive controls were included on each assay plate in order to follow stability over time. The assay is validated for the prototypic SARS-CoV-2 D614G variant and the Omicron BA.5/BA.5 sublineage but has not been validated for the Omicron BQ.1.1 and XBB.1 sublineages. Cell lines used were routinely tested for mycoplasma contamination.

The spike-pseudotyped viruses were derived from ancestral Wuhan-Hu-1 with the following amino acid substitutions: (prototype [D614G]; Omicron subvariants BA.4 and BA.5, designated BA.4/BA.5 for identical spike sequences between BA.4 and BA.5 [T19I, L24S, ΔP25, ΔP26, ΔA27, G142D, V213G, G339D, S371F, S373P, S375F, T376A, D405N, R408S, K417N, N440K, L452R, S477N, T478K, E484A, F486V, Q498R, N501Y, Y505H, D614G, H655Y, N679K, P681G, N764K, D796Y, Q954H, N969K]); Omicron subvariant BQ.1.1 [T19I, L24S, ΔP25, ΔP26, ΔA27, H69-, V70-, G142D, V213G, G339D, R346T, S371F, S373P, S375F, T376A, D405N, R408S, K417N, N440K, K444T, L452R, N460K, S477N, T478K, E484A, F486V, Q498R, N501Y, Y505H, D614G, H655Y, N679K, P681H, N764K, D796Y, Q954H, N969K]; Omicron subvariant XBB.1.5 [T19I, L24S, ΔP25, ΔP26, ΔA27, V83A, G142D, Y145Q, ΔH146, Q183E, V213E, G252V, G339H, R346T, L368I, S371F, S373P, S375F, T376A, D405N, R408S, K417N, N440K, V445P, G446S, N460K, S477N, T478K, E484A, F486P, F490S, Q498R, N501Y, Y505H, D614G, H655Y, N679K, P681H, N764K, D796Y, Q954H, N969K]; Omicron subvariant XBB.1.16 [T19I, L24S, ΔP25, ΔP26, ΔA27, V83A, G142D, Y145Q, ΔH146, E180V, Q183E, V213E, G252V, G339H, R346T, L368I, S371F, S373P, S375F, T376A, D405N, R408S, K417N, N440K, V445P, G446S, N460K, S477N, T478R, E484A, F486P, F490S, Q498R, N501Y, Y505H, D614G, H655Y, N679K, P681H, N764K, D796Y, Q954H, N969K]; Omicron subvariant EG.5.1 [XBB.1.5 + F456L]; Omicron subvariant BA.2.86 [in16MPLF, T19I, R21T, L24-, P25-, P26-, A27S, S50L, H69-, V70-, V127F, G142D, Y144-, F157S, R158G, N211-, L212I, V213G, L216F, H245N, A264D, I332V, G339H, K356T, S371F, S373P, S375F, T376A, R403K, D405N, R408S, K417N, N440K, V445H, G446S, N450D, L452W, N460K, S477N, T478K, N481K, V483-, E484K, F486P, Q498R, N501Y, Y505H, E554K, A570V, D614G, P621S, H655Y, I670V, N679K, P681R, N764K, D796Y, S939F, Q954H, N969K, P1143L]. Omicron subvariant JN.1 [T19I, R21T, L24-, P25-, P26-, A27S, S50L, H69-, V70-, V127F, G142D, Y144-, F157S, R158G, N211-, L212I, V213G, L216F, H245N, A264D, I332V, G339H, K356T, S371F, S373P, S375F, T376A, R403K, D405N, R408S, K417N, N440K, V445H, G446S, N450D, L452W, L455S, N460K, S477N, T478K, N481K, V483-, E484K, F486P, Q498R, N501Y, Y505H, E554K, A570V, D614G, P621S, H655Y, N679K, P681R, N764K, D796Y, S939F, Q954H, N969K, P1143L].

***VSV-based SARS-CoV-2 Pseudovirus Neutralization Assay***

Codon-optimized full-length spike genes (Wuhan-Hu-1 with D614G, BA.4/BA.5, XBB.1.5, XBB.1.16, XBB.2.3.2, EG.5.1, FL.1.5.1, BA.2.86, HK.3.1, HV.1, JN.1) were cloned into a pCAGGS vector. Spike genes contained the following mutations: (a) BA.4/BA.5: T19I, L24- P25- P26-, A27S H69- V70-, G142D, V213G, G339D, S371F, S373P, S375F, T376A, D405N, R408S, K417N, N440K, L452R, S477N, T478K, E484A, F486V, Q498R, N501Y, Y505H, D614G, H655Y, N679K, P681H, N764K, D796Y, Q954H, N969K; (b) XBB.1.5: T19I, L24-, P25-, P26-, A27S, V83A, G142D, Y144-, H146Q, Q183E, V213E, G252V, G339H, R346T, L368I, S371F, S373P, S375F, T376A, D405N, R408S, K417N, N440K, V445P, G446S, N460K, S477N, T478K, E484A, F486P, F490S, Q498R, N501Y, Y505H, D614G, H655Y, N679K, P681H, N764K, D796Y, Q954H, N969K; (c) XBB.1.16: T19I, L24-, P25-, P26-, A27S, V83A, G142D, Y144-, H146Q, E180V, Q183E, V213E, G252V, G339H, R346T, L368I, S371F, S373P, S375F, T376A, D405N, R408S, K417N, N440K, V445P, G446S, N460K, S477N, T478R, E484A, F486P, F490S, Q498R, N501Y, Y505H, D614G, H655Y, N679K, P681H, N764K, D796Y, Q954H, N969K; (d) XBB.2.3.2: T19I, L24-, P25-, P26-, A27S, V83A, G142D, Y144-, H146Q, Q183E, G184V, V213E, D253G, G339H, R346T, L368I, S371F, S373P, S375F, T376A, D405N, R408S, K417N, N440K, V445P, G446S, N460K, S477N, T478K, E484A, F486P, F490S, Q498R, N501Y, Y505H, P521S, D614G, H655Y, N679K, P681H, N764K, D796Y, Q954H, N969K; (e) EG.5.1: T19I L24- P25- P26- A27S Q52H V83A G142D Y144- H146Q Q183E V213E G252V G339H R346T L368I S371F S373P S375F T376A D405N R408S K417N N440K V445P G446S F456L N460K S477N T478K E484A F486P F490S Q498R N501Y Y505H D614G H655Y N679K P681H N764K D796Y Q954H N969K; (f) FL.1.5.1: T19I L24- P25- P26- A27S V83A G142D Y144- H146Q Q183E V213E G252V G339H R346T L368I S371F S373P S375F T376A D405N R408S K417N N440K V445P G446S F456L N460K S477N T478R E484A F486P F490S Q498R N501Y Y505H D614G H655Y N679K P681H A701V N764K D796Y Q954H N969K; (g) BA.2.86: ins16MPLF T19I R21T L24- P25- P26- A27S S50L H69- V70- V127F G142D Y144- F157S R158G N211- L212I V213G L216F H245N A264D I332V G339H K356T S371F S373P S375F T376A R403K D405N R408S K417N N440K V445H G446S N450D L452W N460K S477N T478K N481K V483- E484K F486P Q498R N501Y Y505H E554K A570V D614G P621S H655Y N679K P681R N764K D796Y S939F Q954H N969K P1143L; (h) HK.3.1: T19I L24- P25- P26- A27S Q52H V83A G142D Y144- H146Q F157L Q183E V213E G252V G339H R346T L368I S371F S373P S375F T376A D405N R408S K417N N440K V445P G446S L455F F456L N460K S477N T478K E484A F486P F490S Q498R N501Y Y505H D614G H655Y N679K P681H N764K D796Y Q954H N969K; (i) HV.1: T19I L24- P25- P26- A27S Q52H V83A G142D Y144- H146Q F157L Q183E V213E G252V G339H R346T L368I S371F S373P S375F T376A D405N R408S K417N N440K V445P G446S L452R F456L N460K S477N T478K E484A F486P F490S Q498R N501Y Y505H D614G H655Y N679K P681H N764K D796Y Q954H N969K; (j) JN.1: ins16MPLF T19I R21T L24- P25- P26- A27S S50L H69- V70- V127F G142D Y144- F157S R158G N211- L212I V213G L216F H245N A264D I332V G339H K356T S371F S373P S375F T376A R403K D405N R408S K417N N440K V445H G446S N450D L452W L455S N460K S477N T478K N481K V483- E484K F486P Q498R N501Y Y505H E554K A570V D614G P621S H655Y N679K P681R N764K D796Y S939F Q954H N969K P1143L. To generate VSV∆G-based SARS-CoV-2 pseudovirus, BHK-21/WI-2 cells were transfected with the spike expression plasmid and infected by VSV∆G-firefly-luciferase as previously described (Whitt 2010 Journal of Virological Methods; PMID 20709108). Vero E6 cells (ATCC, CRL-1586) were used as target cells for the neutralization assay and maintained in DMEM supplemented with 10% fetal bovine serum. To perform neutralization assay, serum samples were heat-inactivated for 45 min at 56ºC, and serial dilutions were made in DMEM supplemented with 10% FBS. The diluted serum samples or culture medium (serving as virus only control) were mixed with VSV∆G-based SARS-CoV-2 pseudovirus and incubated at 37ºC for 45 min. The inoculum virus or virus-serum mix was subsequently used to infect Vero E6 cells for 18 h at 37ºC. At 18 h post infection, an equal volume of One-Glo reagent (Promega; E6120) was added to culture medium for readout using BMG PHERAstar FSX plate reader. The percentage of neutralization was calculated based on relative light units of the virus control, and subsequently analyzed using four parameter logistic curve (GraphPad Prism 8.0).

## **Figure S1. Neutralizing Antibody Titers and Geometric Fold-rises Against XBB.1.5, XBB.1.16, BA.4/BA.5 and BQ.1.1 Variants and Ancestral SARS-CoV-2 (D614G) After 3^rd^ Booster Dose of XBB.1.5-containing Monovalent and Bivalent Vaccines by SARS-CoV-2 Status**

**A. All Participants**

**B. No Prior Infection**

**C. Prior Infection**

**Figure S1.** Distribution of neutralizing antibody titers (ID50 log_10_) in the pseudovirus assay against Omicron XBB.1.5, XBB.1.16, BA.4/BA.5, BQ.1.1 and ancestral SARS-CoV-2 (D614G) are shown for serum samples collected before the booster dose of 50-µg of monovalent mRNA-1273.815 or 50-µg of bivalent mRNA-1273.231, at 15 days (Day 15) and at 29 days (Day 29) after the booster dose in the per-protocol set for immunogenicity (with and without prior SARS-CoV-2 infection; Without prior SARS-CoV-2 infection; With prior SARS-CoV-2 infection). The circles are values from individual serum samples. Pseudovirus neutralizing antibody assay lower limits of quantification (LLOQ) are 18.5 (1.3 log_10_) for ancestral SARS-CoV-2 (D614G) and 36.7 (1.6 log_10_) for Omicron BA.4/BA.5; upper limits of quantification (ULOQ) are 45,118 (4.7 log_10_) for ancestral SARS-CoV-2 (D614G) and 13,705 (4.1 log_10_) for Omicron BA.4/BA.5. The Limits of Detection are 10 for XBB.1.5, XBB.1.16 and BQ.1.1. Boxes and horizontal bars denote interquartile ranges (IQR) and median endpoint titers; whisker endpoints are the maximum and minimum values below or above the median ±1.5 times the IQR.

## **Figure S2:** **Neutralizing Antibody Titers and Geometric Fold-rises Against XBB.1.5, XBB.1.16, BA.4/BA.5 and BQ.1.1 Variants and Ancestral SARS-CoV-2 (D614G) After 3^rd^ Booster Dose of XBB.1.5-containing Monovalent and Bivalent Vaccines by Age Groups**

**A. Monovalent mRNA-1273.815**

**B. Bivalent mRNA-1273.815**

**Figure S2.** Distribution of neutralizing antibody titers (ID50 log_10_) in the pseudovirus assay against Omicron XBB.1.5, XBB.1.16, BA.4/BA.5, BQ.1.1 and ancestral SARS-CoV-2 (D614G) are shown for serum samples collected before the booster dose of 50-µg of monovalent mRNA-1273.815 (A) or 50-µg of bivalent mRNA-1273.231 (B), at 15 days (Day 15) and at 29 days (Day 29) after the booster dose in the per-protocol set for immunogenicity (with and without prior SARS-CoV-2 infection) by age (16-64 years; ≥65 years). The circles and lines are values from individual serum samples. Pseudovirus neutralizing antibody assay lower limits of quantification (LLOQ) are 18.5 (1.3 log_10_) for ancestral SARS-CoV-2 (D614G) and 36.7 (1.6 log_10_) for Omicron BA.4/BA.5; upper limits of quantification (ULOQ) are 45,118 (4.7 log_10_) for ancestral SARS-CoV-2 (D614G) and 13,705 (4.1 log_10_) for Omicron BA.4/BA.5. The Limits of Detection are 10 for XBB.1.5, XBB.1.16 and BQ.1.1. Boxes and horizontal bars denote interquartile ranges (IQR) and median endpoint titers; whisker endpoints are the maximum and minimum values below or above the median ±1.5 times the IQR.

## **Figure S3. Analysis of Neutralizing Antibody Titers Against Ancestral SARS-CoV-2 (D614G) and BA.4/BA.5, XBB.1.5, XBB.1.16, XBB.2.3.2, EG.5.1, FL.1.5.1, BA.2.86, JN.1, HK.3.1 and HV.1 Variants in a Randomly-selected Subset of Participants Who Received Monovalent mRNA-1273.815**

**Figure S3.** Analysis of neutralizing antibody titers against variants in 20 randomly-selected all participants and in those with (n=10) and without (n=10) prior SARS-CoV-2 infection in participants who received a booster injection of monovalent mRNA-1273.815 using a research grade VSV-based pseudovirus assay (supplementary methods)**.** Sera were collected before (Pre) and 2 weeks after the administration of mRNA-1273.815 (Post). **p≤0.01 and ****p<0.0001 by the Wilcoxon t-test. Dotted line denotes the limit of detection (20) of the assay.

## **Table S1. Objectives and Endpoints Part J**

| **Objectives** | **Endpoints** |
| --- | --- |
| **Primary** | |
| - To evaluate the immunogenicity of mRNA- 1273.815 (50 µg) and mRNA-1273.231   (50 µg) against SARS-CoV-2 Omicron BA.4/BA.5, BQ.1.1, and XBB.1.5  subvariants at Day 15 and Day 29 | - Antibody response of mRNA-1273.815 (50 µg) and mRNA-1273.231 (50 µg) against   SARS-CoV-2 Omicron BA.4/BA.5, BQ.1.1, and  XBB.1.5 subvariants by GMT, GMFR, and SRR at Day 15 and Day 29 |
| - To evaluate the safety and reactogenicity of mRNA-1273.815 (50 µg) and mRNA-1273.231 (50 µg) | - Solicited local and systemic reactogenicity ARs during a 7-day follow-up period after injection - Unsolicited AEs during the 28-day follow-up period after injection - SAEs, MAAEs, AEs leading to withdrawal, and AESI from Day 1 to Day 181 (EoS) |
| **Exploratory** | |
| - To evaluate the immunogenicity of mRNA- 1273.815 (50 µg) and mRNA-1273.231 (50 µg) against SARS-CoV-2 variants at any study timepoint | - Antibody response of mRNA-1273.815 (50 µg) and mRNA-1273.231 (50 µg) against SARS-CoV-2 variants by GMT, GMFR, and SRR at any study timepoint |
| - To assess for symptomatic and asymptomatic SARS-CoV-2 infection | - Laboratory-confirmed symptomatic or asymptomatic SARS-CoV-2 infection will be defined in participants: - Primary case definition per the mRNA-1273-P301 (COVE) study - Secondary case definition based on the CDC criteria: the presence of 1 of the CDC-listed symptoms (CDC 2022) and a positive RT-PCR test on a respiratory sample - Asymptomatic SARS-CoV-2 infection is defined as a positive RT-PCR test on a respiratory sample in the absence of symptoms or a positive serologic test for antinucleocapsid antibody after a negative test at time of enrollment |

## **Table S2. Neutralizing Antibodies Titers and Geometric-fold Rises After 3rd Booster Dose of XBB.1.5-Containing Monovalent and Bivalent Vaccines Against XBB.1.5, XBB.1.16, BA.4/BA.5, BQ.1.1, EG.5.1, BA.2.86 and JN.1 Variants and Ancestral SARS-CoV-2 (D614G), Per-protocol Immunogenicity Set**

|  | **Monovalent mRNA-1273.815**  **50 µg** | **Bivalent mRNA-1273.231**  **50 µg** |
| --- | --- | --- |
|  | **N=49** | **N=50** |
| **XBB.1.5** | | |
| Pre-booster, n | 49 | 50 |
| GM Level (95% CI) | 154.7 (106.8-224.1) | 158.8 (109.9-229.3) |
| Day 15, n | 49 | 50 |
| GM Level (95% CI) | 2579.0 (1809.1-3676.7) | 1838.1 (1265.9-2668.9) |
| GMFR (95% CI) | 16.7 (12.8-21.7) | 11.6 (8.7-15.4) |
| Day 29, n | 49 | 50 |
| GM Level (95% CI) | 2710.6 (1778.3-4131.8) | 2059.5 (1440.2-2945.3) |
| GMFR (95% CI) | 17.5 (13.0-23.6) | 13.0 (9.9-17.0) |
| **XBB.1.16** | | |
| Pre-booster, n | 47 | 50 |
| GM Level (95% CI) | 221.0 (153.9-317.3) | 193.9 (134.1-280.3) |
| Day 15, n | 49 | 50 |
| GM Level (95% CI) | 2262.6 (1570.1-3260.6) | 1799.9 (1297.2-2497.5) |
| GMFR (95% CI) | 11.4 (8.5-15.4) | 9.3 (7.0-12.3) |
| Day 29, n | 49 | 50 |
| GM Level (95% CI) | 3694.3 (2364.2-5772.7) | 2386.3 (1632.5-3488.3) |
| GMFR (95% CI) | 19.0 (13.7-26.5) | 12.3 (9.3-16.3) |
| **BA.4/BA.5** |  |  |
| Pre-booster, n | 49 | 50 |
| GM Level (95% CI) | 1540.7 (1127.2-2105.8) | 1878.1 (1350.9-2611.1) |
| Day 15, n | 49 | 50 |
| GM Level (95% CI) | 9673.4 (6965.6-13433.8) | 9904.8 (7610.8-12890.1) |
| GMFR (95% CI) | 6.3 (4.8-8.2) | 5.3 (3.9-7.1) |
| Day 29, n | 49 | 50 |
| GM Level (95% CI) | 11231.0 (7883.5-15999.9) | 11037.0 (8090.8-15055.9) |
| GMFR (95% CI) | 7.3 (5.4-9.9) | 5.9 (4.4-7.8) |
| **BQ.1.1** |  |  |
| Pre-booster, n | 48 | 50 |
| GM Level (95% CI) | 347.5 (249.5-483.9) | 312.8 (221.4-441.9) |
| Day 15, n | 49 | 50 |
| GM Level (95% CI) | 1894.1 (1383.2-2593.6) | 1895.4 (1348.2-2664.7) |
| GMFR (95% CI) | 5.8 (4.7-7.3) | 6.1 (4.6-7.9) |
| Day 29, n | 48 | 50 |
| GM Level (95% CI) | 2255.6 (1599.4-3181.1) | 1790.1 (1336.1-2398.4) |
| GMFR (95% CI) | 6.9 (5.5-8.6) | 5.7 (4.4-7.4) |
| **Ancestral SARS-CoV-2 (D614G)** | | |
| Pre-booster, n | 49 | 50 |
| GM Level (95% CI) | 2780.3 (2146.5-3601.3) | 2413.4 (1783.3-3266.1) |
| Day 15, n | 49 | 50 |
| GM Level (95% CI) | 7749.7 (5943.7-10104.3) | 5937.9 (4635.5-7606.2) |
| GMFR (95% CI) | 2.8 (2.2-3.5) | 2.5 (1.9-3.1) |
| Day 29, n | 49 | 50 |
| GM Level (95% CI) | 12191.4 (9089.2-16352.5) | 9066.5 (6916.3-11885.1) |
| GMFR (95% CI) | 4.4 (3.5-5.5) | 3.8 (3.0-4.7) |
| **EG.5.1** | | |
| Pre-booster, n | 49 | NA |
| GM Level (95% CI) | 73.8 (50.5-107.9) | NA |
| Day 15, n | NA | NA |
| GM Level (95% CI) | NA | NA |
| GMFR (95% CI) | NA | NA |
| Day 29, n | 46 | NA |
| GM Level (95% CI) | 1041.8 (681.0-1593.8) | NA |
| GMFR (95% CI) | 14.8 (11.0-19.9) | NA |
| **BA.2.86** | | |
| Pre-booster, n | 47 | NA |
| GM Level (95% CI) | 104.0 (71.7-150.9) | NA |
| Day 15, n | NA | NA |
| GM Level (95% CI) | NA | NA |
| GMFR (95% CI) | NA | NA |
| Day 29, n | 49 | NA |
| GM Level (95% CI) | 1076.7 (733.2-1581.1) | NA |
| GMFR (95% CI) | 10.4 (8.2-13.2) | NA |
| **JN.1** | | |
| Pre-booster, n | 47 | NA |
| GM Level (95% CI) | 40.9 (28.3-59.2) | NA |
| Day 15, n | NA | NA |
| GM Level (95% CI) | NA | NA |
| GMFR (95% CI) | NA | NA |
| Day 29, n | 49 | NA |
| GM Level (95% CI) | 466.4 (302.8-718.6) | NA |
| GMFR (95% CI) | 11.2 (8.7-14.3) | NA |
| n = number of observations at corresponding timepoint; GM = geometric mean; 95% CI = 95% confidence interval; GMFR = geometric mean fold rise; LLOQ=lower limit of quantification; ULOQ=upper limit of quantification; NA=Not available. 95% CI is calculated based on the t-distribution of the log-transformed values or the difference in the log-transformed values for GM value and GM fold-rise, respectively, then back transformed to the original scale for presentation. GMFR is GM Level at Day 15 relative to GM Level at pre-booster. Antibody values reported as below the LLOQ (36.7 for BA.4/BA.5; 18.5 for ancestral SARS-CoV-2 [D614G]) are replaced by 0.5 x LLOQ. Values greater than the ULOQ (13,705 for BA.4/BA.5; 45,118 for Ancestral SARS-CoV-2 (D614G)) are replaced by the ULOQ if actual values are not available. Antibody values reported as below the lower limit of detection (LOD 10 for XBB.1.5, XBB.1.16, BQ.1.1, EG.5, BA.2.86 and JN.1) are replaced by 0.5 x LOD. | | |

## **Table S3. Neutralizing Antibodies Titers and Geometric-fold Rises After 3rd Booster Dose of XBB.1.5-Containing Monovalent and Bivalent Vaccines Against XBB.1.5, XBB.1.16, BA.4/BA.5, BQ.1.1, EG.5.1, BA.2.86 and JN.1 Variants and Ancestral SARS-CoV-2 (D614G) by Pre-booster SARS-CoV-2 Status**

|  | **No prior SARS-CoV-2 infection** | | **Prior SARS-CoV-2 infection** | |
| --- | --- | --- | --- | --- |
|  | **Monovalent**  **mRNA-1273.815**  **N=16** | **Bivalent**  **mRNA-1273.231**  **N=11** | **Monovalent**  **mRNA-1273.815**  **N=33** | **Bivalent**  **mRNA-1273.231**  **N=39** |
| **XBB.1.5** | | | | |
| Pre-booster, n | 16 | 11 | 33 | 39 |
| GM Level (95% CI) | 81.7 (40.7-164.1) | 65.2 (33.8-125.6) | 210.8 (138.8-320.3) | 204.1 (135.3-307.9) |
| Day 15, n | 16 | 11 | 33 | 39 |
| GM Level (95% CI) | 1639.3 (742.9-3617.4) | 1256.9 (537.5-2939.4) | 3212.8 (2215.2-4659.5) | 2046.1 (1333.2-3140.1) |
| GMFR (95% CI) | 20.1 (12.5-32.1) | 19.3 (8.5-43.8) | 15.2 (10.9-21.2) | 10.0 (7.5-13.5) |
| Day 29, n | 16 | 11 | 33 | 39 |
| GM Level (95% CI) | 1543.1 (644.3-3695.6) | 1431.4 (606.0-3381.4) | 3562.0 (2232.5-5683.2) | 2282.1 (1521.7-3422.5) |
| GMFR (95% CI) | 18.9 (11.3-31.5) | 22.0 (11.8-40.8) | 16.9 (11.5-24.9) | 11.2 (8.3-15.1) |
| **XBB.1.16** | | | | |
| Pre-booster, n | 14 | 11 | 33 | 39 |
| GM Level (95% CI) | 133.7 (67.9-263.2) | 71.6 (33.9-150.8) | 273.5 (178.0-420.1) | 256.9 (173.3-380.7) |
| Day 15, n | 16 | 11 | 33 | 39 |
| GM Level (95% CI) | 1409.5 (646.1-3074.8) | 1452.8 (618.3-3413.6) | 2846.2 (1912.7-4235.4) | 1912.0 (1326.8-2755.2) |
| GMFR (95% CI) | 14.2 (8.5-24.0) | 20.3 (9.6-43.0) | 10.4 (7.2-15.1) | 7.4 (5.6-9.8) |
| Day 29, n | 16 | 11 | 33 | 39 |
| GM Level (95% CI) | 2246.4 (840.7-6002.2) | 1743.7 (712.0-4270.1) | 4702.0 (2905.3-7610.0) | 2607.1 (1688.3-4026.1) |
| GMFR (95% CI) | 24.1 (13.1-44.4) | 24.4 (12.4-47.7) | 17.2 (11.4-25.9) | 10.2 (7.5-13.7) |
| **BA.4/BA.5** | | | | |
| Pre-booster, n | 16 | 11 | 33 | 39 |
| GM Level (95% CI) | 691.3 (438.9-1088.7) | 844.4 (405.9-1756.9) | 2272.3 (1606.0-3215.0) | 2353.1 (1653.9-3347.9) |
| Day 15, n | 16 | 11 | 33 | 39 |
| GM Level (95% CI) | 6254.4 (3482.3-11233.1) | 9269.3 (6282.1-13676.8) | 11951.0 (8035.2-17775.2) | 10091.8 (7276.3-13996.6) |
| GMFR (95% CI) | 9.0 (5.9-13.9) | 11.0 (4.5-27.0) | 5.3 (3.7-7.4) | 4.3 (3.3-5.6) |
| Day 29, n | 16 | 11 | 33 | 39 |
| GM Level (95% CI) | 7720.5 (3771.2-15805.6) | 9415.3 (5412.0-16379.8) | 13469.1 (8954.7-20259.4) | 11542.9 (7918.9-16825.4) |
| GMFR (95% CI) | 11.2 (6.4-19.4) | 11.1 (5.2-23.9) | 5.9 (4.1-8.6) | 4.9 (3.6-6.6) |
| **BQ.1.1** | | | | |
| Pre-booster, n | 15 | 11 | 33 | 39 |
| GM Level (95% CI) | 247.7 (125.7-488.2) | 142.2 (71.7-281.9) | 405.3 (275.9-595.3) | 390.7 (266.1-573.5) |
| Day 15, n | 16 | 11 | 33 | 39 |
| GM Level (95% CI) | 1438.3 (696.2-2971.2) | 1376.1 (877.5-2157.8) | 2164.5 (1554.6-3013.6) | 2074.6 (1360.0-3164.5) |
| GMFR (95% CI) | 7.1 (4.6-11.1) | 9.7 (4.5-20.9) | 5.3 (4.1-7.0) | 5.3 (4.0-7.0) |
| Day 29, n | 15 | 11 | 33 | 39 |
| GM Level (95% CI) | 1661.3 (699.9-3943.0) | 1590.7 (1041.3-2430.0) | 2592.0 (1841.5-3648.5) | 1850.7 (1286.9-2661.6) |
| GMFR (95% CI) | 8.2 (5.1-13.1) | 11.2 (6.0-20.8) | 6.4 (5.0-8.3) | 4.7 (3.6-6.2) |
| **Ancestral SARS-CoV-2 (D614G)** | | | | |
| Pre-booster, n | 16 | 11 | 33 | 39 |
| GM Level (95% CI) | 2179.9 (1205.3-3942.6) | 1746.1 (814.6-3742.8) | 3128.4 (2380.6-4111.1) | 2644.0 (1886.7-3705.3) |
| Day 15, n | 16 | 11 | 33 | 39 |
| GM Level (95% CI) | 7743.5 (4611.2-13003.4) | 6570.7 (4211.1-10252.5) | 7752.7 (5605.8-10721.9) | 5770.7 (4272.7-7793.9) |
| GMFR (95% CI) | 3.6 (2.1-5.9) | 3.8 (1.7-8.4) | 2.5 (2.0-3.1) | 2.2 (1.7-2.7) |
| Day 29, n | 16 | 11 | 33 | 39 |
| GM Level (95% CI) | 12825.9 (6736.8-24419.0) | 11970.6 (7757.7-18471.3) | 11895.2 (8527.0-16593.7) | 8383.0 (6031.3-11651.7) |
| GMFR (95% CI) | 5.9 (3.6-9.7) | 6.9 (3.7-12.6) | 3.8 (3.0-4.8) | 3.2 (2.5-4.0) |
| **EG.5.1** | | | | |
| Pre-booster, n | 16 | NA | 33 | NA |
| GM Level (95% CI) | 43.2 (20.6-90.7) | NA | 95.7 (62.1-147.7) | NA |
| Day 15, n | NA | NA | NA | NA |
| GM Level (95% CI) | NA | NA | NA | NA |
| GMFR (95% CI) | NA | NA | NA | NA |
| Day 29, n | 15 | NA | 31 | NA |
| GM Level (95% CI) | 628.0 (276.0-1428.7) | NA | 1331.0 (807.5-2193.8) | NA |
| GMFR (95% CI) | 16.2 (9.1-28.9) | NA | 14.2 (9.9-20.4) | NA |
| **BA.2.86** | | | | |
| Pre-booster, n | 15 | NA | 32 | NA |
| GM Level (95% CI) | 60.0 (30.8-116.5) | NA | 134.6 (86.4-209.9) | NA |
| Day 15, n | NA | NA | NA | NA |
| GM Level (95% CI) | NA | NA | NA | NA |
| GMFR (95% CI) | NA | NA | NA | NA |
| Day 29, n | 16 | NA | 33 | NA |
| GM Level (95% CI) | 725.5 (322.2-1633.6) | NA | 1303.8 (846.9-2007.2) | NA |
| GMFR (95% CI) | 13.1 (8.5-20.0) | NA | 9.3 (6.9-12.5) | NA |
| **JN.1** | | | | |
| Pre-booster, n | 15 | NA | 32 | NA |
| GM Level (95% CI) | 25.0 (12.9-48.3) | NA | 51.6 (33.1-80.5) | NA |
| Day 15, n | NA | NA | NA | NA |
| GM Level (95% CI) | NA | NA | NA | NA |
| GMFR (95% CI) | NA | NA | NA | NA |
| Day 29, n | 16 | NA | 33 | NA |
| GM Level (95% CI) | 288.5 (113.2-735.3) | NA | 588.8 (366.9-944.8) | NA |
| GMFR (95% CI) | 11.9 (6.7-21.3) | NA | 10.8 (8.2-14.3) | NA |
| n=number of participants with non-missing data at the timepoint (pre-booster or Day 29), CI=confidence interval, GM=geometric mean, GMFR=geometric mean fold rise (day 29 compared with pre-booster), LLOQ=lower limit of quantification, ULOQ=upper limit of quantification; NA=Not available. Antibody values assessed by pseudovirus neutralizing antibody assay reported as below the LLOQ (36.7 for BA.4/BA.5; 18.5 for Ancestral SARS-CoV-2 [D614G]) are replaced by 0.5 × LLOQ. Values greater than ULOQ (13,705 for BA.4/BA.5; 45,118 for Ancestral SARS-CoV-2) are replaced by the ULOQ if actual values are not available. Antibody values reported as below the lower limit of detection (LOD 10 for XBB.1.5, XBB.1.16, BQ.1.1, EG.5. BA.2.86 and JN.1) are replaced by 0.5 x LOD. 95% CI is calculated based on the t-distribution of the log-transformed values or the difference in the log-transformed values for GM Level and GMFR, respectively, then back transformed to the original scale. | | | | |

## **Table S4.** **Neutralizing Antibodies Titers and Geometric Fold-rises Against XBB.1.5, XBB.1.16, BA.4/BA.5 and BQ.1.1 Variants and Ancestral SARS-CoV-2 (D614G) After 3^rd^ Booster Dose of XBB.1.5-Containing Monovalent and Bivalent Vaccines by Age Groups**

|  | **18 to <65 Years** | | **≥65 Years** | |
| --- | --- | --- | --- | --- |
|  | **Monovalent**  **mRNA-1273.815**  **N=38** | **Bivalent**  **mRNA-1273.231**  **N=43** | **Monovalent**  **mRNA-1273.815**  **N=11** | **Bivalent**  **mRNA-1273.231**  **N=7** |
| **XBB.1.5** | | | | |
| Pre-booster, n | 38 | 43 | 11 | 7 |
| GM Level (95% CI) | 132.0 (90.4-192.9) | 158.3 (108.7-230.6) | 267.5 (89.8-797.1) | 161.6 (30.9-845.3) |
| Day 15, n | 38 | 43 | 11 | 7 |
| GM Level (95% CI) | 2035.8 (1370.2-3024.8) | 1879.9 (1280.3-2760.3) | 5838.3 (2982.4-11429.0) | 1600.8 (311.6-8225.1) |
| GMFR (95% CI) | 15.4 (11.8-20.2) | 11.9 (8.5-16.5) | 21.8 (9.6-49.4) | 9.9 (6.0-16.2) |
| Day 29, n | 38 | 43 | 11 | 7 |
| GM Level (95% CI) | 1998.3 (1249.9-3194.9) | 2063.3 (1420.9-2996.2) | 7771.3 (3696.3-16338.6) | 2036.4 (451.2-9191.2) |
| GMFR (95% CI) | 15.1 (11.2-20.4) | 13.0 (9.6-17.7) | 29.1 (11.9-71.1) | 12.6 (6.7-23.7) |
| **XBB.1.16** | | | | |
| Pre-booster, n | 37 | 43 | 10 | 7 |
| GM Level (95% CI) | 183.5 (123.6-272.3) | 192.0 (133.9-275.4) | 439.5 (183.6-1052.2) | 205.6 (31.5-1340.9) |
| Day 15, n | 38 | 43 | 11 | 7 |
| GM Level (95% CI) | 1759.3 (1168.1-2649.7) | 1816.6 (1307.6-2523.8) | 5395.9 (2800.8-10395.5) | 1700.5 (356.5-8111.3) |
| GMFR (95% CI) | 10.6 (7.8-14.4) | 9.5 (6.9-13.0) | 14.9 (5.7-39.0) | 8.3 (3.9-17.5) |
| Day 29, n | 38 | 43 | 11 | 7 |
| GM Level (95% CI) | 2621.3 (1623.2-4233.2) | 2381.7 (1608.4-3526.6) | 12087.3 (4995.2-29248.3) | 2415.2 (463.4-12588.4) |
| GMFR (95% CI) | 15.9 (11.4-22.3) | 12.4 (9.1-17.0) | 36.8 (14.1-95.8) | 11.7 (5.2-26.6) |
| **BA.4/BA.5** | | | | |
| Pre-booster, n | 38 | 43 | 11 | 7 |
| GM Level (95% CI) | 1396.7 (987.8-1974.8) | 1838.1 (1293.0-2612.9) | 2162.3 (973.9-4800.8) | 2144.1 (618.2-7436.8) |
| Day 15, n | 38 | 43 | 11 | 7 |
| GM Level (95% CI) | 8256.7 (5637.0-12093.7) | 9986.6 (7551.3-13207.4) | 16717.1 (8962.2-31182.4) | 9416.4 (3363.6-26361.3) |
| GMFR (95% CI) | 5.9 (4.4-8.0) | 5.4 (3.9-7.6) | 7.7 (3.9-15.3) | 4.4 (2.2-8.9) |
| Day 29, n | 38 | 43 | 11 | 7 |
| GM Level (95% CI) | 8932.8 (6069.9-13146.1) | 10961.5 (7903.8-15202.0) | 24768.7 (11560.0-53070.0) | 11512.2 (3280.9-40395.1) |
| GMFR (95% CI) | 6.4 (4.5-9.0) | 6.0 (4.3-8.3) | 11.5 (5.6-23.5) | 5.4 (2.6-10.9) |
| **BQ.1.1** | | | | |
| Pre-booster, n | 37 | 43 | 11 | 7 |
| GM Level (95% CI) | 304.1 (212.5-435.1) | 302.5 (206.5-443.1) | 544.5 (228.6-1296.6) | 384.0 (137.0-1076.1) |
| Day 15, n | 38 | 43 | 11 | 7 |
| GM Level (95% CI) | 1503.9 (1075.9-2102.0) | 1848.7 (1297.9-2633.2) | 4202.3 (2140.7-8249.5) | 2209.7 (519.0-9408.9) |
| GMFR (95% CI) | 5.4 (4.1-7.0) | 6.1 (4.5-8.3) | 7.7 (5.2-11.5) | 5.8 (3.7-8.8) |
| Day 29, n | 38 | 43 | 10 | 7 |
| GM Level (95% CI) | 1728.1 (1204.5-2479.4) | 1691.5 (1237.7-2311.6) | 6207.5 (3120.8-12347.2) | 2535.5 (891.5-7211.2) |
| GMFR (95% CI) | 6.3 (4.9-7.9) | 5.6 (4.1-7.5) | 9.8 (5.5-17.6) | 6.6 (4.2-10.3) |
| **Ancestral SARS-CoV-2 (D614G)** | | | | |
| Pre-booster, n | 38 | 43 | 11 | 7 |
| GM Level (95% CI) | 2571.3 (1985.1-3330.6) | 2450.2 (1742.4-3445.7) | 3642.0 (1600.2-8289.4) | 2198.9 (1041.2-4643.9) |
| Day 15, n | 38 | 43 | 11 | 7 |
| GM Level (95% CI) | 6491.2 (4845.6-8695.8) | 5967.4 (4566.8-7797.5) | 14293.4 (8364.8-24423.8) | 5760.0 (2384.0-13916.7) |
| GMFR (95% CI) | 2.5 (2.0-3.2) | 2.4 (1.8-3.2) | 3.9 (2.1-7.5) | 2.6 (1.7-4.1) |
| Day 29, n | 38 | 43 | 11 | 7 |
| GM Level (95% CI) | 9757.1 (7273.4-13088.8) | 8965.7 (6654.5-12079.7) | 26316.6 (12524.9-55295.0) | 9710.7 (4171.1-22607.5) |
| GMFR (95% CI) | 3.8 (3.1-4.7) | 3.7 (2.8-4.8) | 7.2 (3.7-14.3) | 4.4 (3.1-6.2) |
| n=number of participants with non-missing data at the timepoint (pre-booster or Day 29), CI=confidence interval, GM=geometric mean, GMFR=geometric mean fold rise (day 29 compared with pre-booster), LLOQ=lower limit of quantification, ULOQ=upper limit of quantification. Antibody values assessed by pseudovirus neutralizing antibody assay reported as below the LLOQ (36.7 for BA.4/BA.5; 18.5 for Ancestral SARS-CoV-2 [D614G]) are replaced by 0.5 × LLOQ. Values greater than ULOQ (13,705 for BA.4/BA.5; 45,118 for Ancestral SARS-CoV-2) are replaced by the ULOQ if actual values are not available. Antibody values reported as below the lower limit of detection (LOD 10 for XBB.1.5, XBB.1.16; 10, and BQ.1.1) are replaced by 0.5 x LOD. 95% CI is calculated based on the t-distribution of the log-transformed values or the difference in the log-transformed values for GM Level and GMFR, respectively, then back transformed to the original scale. | | | | |

## **Table S5. Solicited Local and Systemic Adverse Reactions (Solicited Safety Set)**

| **Solicited Adverse Reaction** | **Monovalent mRNA-1273.815**  **50 µg**  **(N=50)** | **Bivalent mRNA-1273.231**  **50 µg**  **(N=51)** | **mRNA-1273**  **50 µg**  **(N=350)** |
| --- | --- | --- | --- |
| Solicited ARs - N1 | 50 | 51 | 350 |
| Any Solicited | 38 (76.0) | 45 (88.2) | 300 (85.7) |
| Grade 1 | 23 (46.0) | 28 (54.9) | 184 (52.6) |
| Grade 2 | 14 (28.0) | 11 (21.6) | 88 (25.1) |
| Grade 3 | 1 (2.0) | 6 (11.8) | 28 (8.0) |
| Grade 4 | 0 | 0 | 0 |
| Local ARs - N1 | 50 | 51 | 350 |
| Any Local | 34 (68.0) | 43 (84.3) | 278 (79.4) |
| Grade 1 | 26 (52.0) | 33 (64.7) | 239 (68.3) |
| Grade 2 | 8 (16.0) | 7 (13.7) | 27 (7.7) |
| Grade 3 | 0 | 3 (5.9) | 12 (3.4) |
| Pain - N1 | 50 | 51 | 350 |
| Any | 34 (68.0) | 42 (82.4) | 268 (76.6) |
| Grade 1 | 28 (56.0) | 33 (64.7) | 240 (68.6) |
| Grade 2 | 6 (12.0) | 7 (13.7) | 24 (6.9) |
| Grade 3 | 0 | 2 (3.9) | 4 (1.1) |
| Erythema (Redness) - N1 | 50 | 51 | 350 |
| Any | 2 (4.0) | 1 (2.0) | 13 (3.7) |
| Grade 1 | 1 (2.0) | 0 | 5 (1.4) |
| Grade 2 | 1 (2.0) | 0 | 6 (1.7) |
| Grade 3 | 0 | 1 (2.0) | 2 (0.6) |
| Swelling (Hardness) - N1 | 50 | 51 | 350 |
| Any | 5 (10.0) | 6 (11.8) | 22 (6.3) |
| Grade 1 | 2 (4.0) | 3 (5.9) | 13 (3.7) |
| Grade 2 | 3 (6.0) | 2 (3.9) | 4 (1.1) |
| Grade 3 | 0 | 1 (2.0) | 5 (1.4) |
| Axillary Swelling or Tenderness - N1 | 50 | 51 | 350 |
| Any | 8 (16.0) | 14 (27.5) | 53 (15.1) |
| Grade 1 | 8 (16.0) | 12 (23.5) | 45 (12.9) |
| Grade 2 | 0 | 2 (3.9) | 4 (1.1) |
| Grade 3 | 0 | 0 | 4 (1.1) |
| Systemic ARs - N1 | 50 | 51 | 350 |
| Any Systemic | 29 (58.0) | 33 (64.7) | 231 (66.0) |
| Grade 1 | 16 (32.0) | 17 (33.3) | 124 (35.4) |
| Grade 2 | 12 (24.0) | 12 (23.5) | 91 (26.0) |
| Grade 3 | 1 (2.0) | 4 (7.8) | 16 (4.6) |
| Fever - N1 | 50 | 51 | 350 |
| Any | 3 (6.0) | 1 (2.0) | 11 (3.1) |
| Grade 1 | 1 (2.0) | 1 (2.0) | 8 (2.3) |
| Grade 2 | 1 (2.0) | 0 | 3 (0.9) |
| Grade 3 | 1 (2.0) | 0 | 0 |
| Headache - N1 | 50 | 51 | 349 |
| Any | 17 (34.0) | 23 (45.1) | 143 (41.0) |
| Grade 1 | 14 (28.0) | 17 (33.3) | 111 (31.8) |
| Grade 2 | 3 (6.0) | 5 (9.8) | 30 (8.6) |
| Grade 3 | 0 | 1 (2.0) | 2 (0.6) |
| Fatigue - N1 | 50 | 51 | 349 |
| Any | 22 (44.0) | 25 (49.0) | 179 (51.3) |
| Grade 1 | 12 (24.0) | 14 (27.5) | 94 (26.9) |
| Grade 2 | 10 (20.0) | 9 (17.6) | 74 (21.2) |
| Grade 3 | 0 | 2 (3.9) | 11 (3.2) |
| Myalgia - N1 | 50 | 51 | 349 |
| Any | 19 (38.0) | 19 (37.3) | 134 (38.4) |
| Grade 1 | 14 (28.0) | 11 (21.6) | 68 (19.5) |
| Grade 2 | 5 (10.0) | 6 (11.8) | 53 (15.2) |
| Grade 3 | 0 | 2 (3.9) | 13 (3.7) |
| Arthralgia - N1 | 50 | 51 | 349 |
| Any | 14 (28.0) | 15 (29.4) | 110 (31.5) |
| Grade 1 | 12 (24.0) | 8 (15.7) | 69 (19.8) |
| Grade 2 | 2 (4.0) | 6 (11.8) | 38 (10.9) |
| Grade 3 | 0 | 1 (2.0) | 3 (0.9) |
| Nausea/Vomiting - N1 | 50 | 51 | 349 |
| Any | 4 (8.0) | 5 (9.8) | 35 (10.0) |
| Grade 1 | 4 (8.0) | 3 (5.9) | 27 (7.7) |
| Grade 2 | 0 | 2 (3.9) | 8 (2.3) |
| Grade 3 | 0 | 0 | 0 |
| Chills - N1 | 50 | 51 | 349 |
| Any | 7 (14.0) | 12 (23.5) | 74 (21.2) |
| Grade 1 | 5 (10.0) | 9 (17.6) | 46 (13.2) |
| Grade 2 | 2 (4.0) | 3 (5.9) | 27 (7.7) |
| Grade 3 | 0 | 0 | 1 (0.3) |
| N1=Number of exposed subjects with data for the event. Any = Grade 1 or higher. Percentages are based on the number of exposed subjects who submitted any data for the event (N1). Toxicity grade for Erythema (Redness) is defined as: G1 = 25 — 50 mm; G2 = 51 — 100 mm; G3 = > 100 mm. Toxicity grade for Fever is defined as: G1 = 38 — 38.4 C; G2 = 38.5 — 38.9 C; G3 = 39 — 40 C; G4 = > 40 C. | | | |
|  | | |  |

## **Table S6. Unsolicited Adverse Events up to 28 Days after the Injection (Safety Set)**

| **n (%)** | **Monovalent mRNA-1273.815**  **50 µg** **(N=50)** | **Bivalent mRNA-1273.231**  **50 µg** **(N=51)** | **mRNA-1273**  **50 µg**  **(N=376)** |
| --- | --- | --- | --- |
| Unsolicited AEs Regardless of Relationship to Study Vaccination | | | |
| All | 6 (12.0) | 9 (17.6) | 80 (21.3) |
| Serious | 0 | 0 | 1 (0.3) |
| Fatal | 0 | 0 | 0 |
| Medically-Attended | 5 (10.0) | 6 (11.8) | 57 (15.2) |
| Leading to study Discontinuation | 0 | 0 | 0 |
| Severe | 0 | 0 | 3 (0.8) |
| Unsolicited AEs Related to Study Vaccination | | | |
| All | 1 (2.0) | 3 (5.9) | 21 (5.6) |
| Serious | 0 | 0 | 0 |
| Fatal | 0 | 0 | 0 |
| Medically-Attended | 1 (2.0) | 0 | 2 (0.5)* |
| Leading to study Discontinuation | 0 | 0 | 0 |
| Severe | 0 | 0 | 2 (0.5) |
| AEs = adverse reactions. AE=adverse event. An adverse event was defined as any event not present prior to study vaccination or any event already present that worsened in intensity or frequency after vaccination.  *Medically attended AEs of hypertension and urticaria (both grade 1) in the mRNA-1273 group. Percentages are based on the number of participants in the safety set. | | | |

## **Supplement References**

1. Gilbert PB, Montefiori DC, McDermott AB, et al. Immune correlates analysis of the mRNA-1273 COVID-19 vaccine efficacy clinical trial. Science 2022;375:43-50.
